# Biomarker-guided immunotherapy in osteosarcoma: from immune microenvironment profiling to patient stratification and combination strategies

**DOI:** 10.3389/fmed.2026.1987146

**Published:** 2026-10-09

**Authors:** Qingqing Li

**Affiliations:** Department of Orthopaedics, Zhejiang Hospital, Hangzhou, Zhejiang, China

**Keywords:** biomarkers, CAR T cells, circulating tumor DNA, immune checkpoint blockade, immunotherapy, osteosarcoma, patient stratification, tumor immune microenvironment

## Abstract

Osteosarcoma remains a therapeutically difficult malignancy, particularly after metastatic relapse, and immune checkpoint blockade or adoptive cell therapy has not reproduced the durable responses achieved in more immunogenic cancers. The central translational problem is not a complete absence of immune activity, but marked interpatient, intratumoral, and spatial heterogeneity in immune access, antigen presentation, myeloid suppression, stromal exclusion, and target-antigen expression. This Mini Review synthesizes recent single-cell and spatial profiling studies with tissue, circulating, and clinical biomarker evidence to define a practical framework for biomarker-guided immunotherapy. Osteosarcoma ecosystems can contain lymphoid-excluded, myeloid-dominant, angiogenic-hypoxic, checkpoint-rich, and antigen-high states that may coexist within the same patient. Consequently, programmed death-ligand 1 (PD-L1) alone is unlikely to function as a reliable treatment-selection marker. A composite assessment integrating human leukocyte antigen (HLA) class I and antigen-processing competence, CD8/regulatory T-cell topology, macrophage and myeloid-derived suppressor cell programs, vascular and extracellular-matrix barriers, and quantitative surface-antigen density is more biologically defensible. Dynamic measurements, including circulating tumor DNA, soluble B7-H3, exosomal immune signals, and serial imaging, may complement tissue profiling but remain investigational. Clinical experience with checkpoint inhibitors, antiangiogenic combinations, mifamurtide, HER2-directed approaches, and emerging B7-H3-, GD2-, ALPL-1-, CD166-, IL-11Ralpha-, and NKG2D-based cellular therapies underscores the need to match mechanism to phenotype. Prospective studies should therefore use standardized, spatially resolved, and longitudinal biomarker panels to enrich trials, distinguish prognostic from predictive effects, and adapt therapy when immune exclusion or antigen escape emerges.

## 1 Introduction

High-grade osteosarcoma is treated with complete surgery and multiagent chemotherapy, yet outcomes have changed little for patients with unresectable, metastatic, or recurrent disease (1). The disease is characterized by chromosomal instability, structural variation, and marked interpatient and intratumoral heterogeneity, which complicate conventional molecular targeting and immune recognition (2, 3). Immunotherapy is therefore attractive but mechanistically demanding: an immune effector must reach mineralized and frequently hypoxic tissue, remain functional in a myeloid-rich microenvironment, and recognize antigens that are sufficiently abundant and homogeneous without unacceptable normal-tissue toxicity.

Biomarker terminology is often used imprecisely in this setting. A prognostic biomarker identifies outcome independent of treatment; a predictive biomarker identifies differential benefit from a specific intervention; a target-eligibility biomarker establishes whether a therapeutic antigen is present at a biologically relevant level; and a pharmacodynamic biomarker shows that treatment has altered its intended pathway. These categories should not be conflated. For example, a marker associated with metastasis or survival does not automatically select patients for checkpoint blockade, and detectable antigen staining does not by itself establish suitability for a chimeric antigen receptor (CAR) T-cell product. For clarity, HLA class I and the antigen-processing machinery determine whether intracellular antigens are displayed to CD8 T cells, whereas T-cell exhaustion denotes loss of effector function during persistent antigen stimulation. Regulatory T cells (Tregs), tumor-associated macrophages (TAMs), and myeloid-derived suppressor cells (MDSCs) can restrain antitumor immunity, while extracellular-matrix (ECM)-mediated exclusion describes stromal or osteoid barriers that limit immune-cell access.

The available evidence does not support one universal biomarker for osteosarcoma immunotherapy. Treatment sensitivity is more plausibly determined by an ecological configuration linking tumor-cell antigenicity, antigen-presentation competence, immune-cell location and function, stromal and vascular accessibility, and the chosen therapeutic platform. This Mini Review integrates recent single-cell and spatial studies, translational biomarker analyses, and clinical trials to propose a biomarker-guided framework for patient stratification and rational combination therapy. The framework is investigational and is intended to generate testable enrichment hypotheses rather than a current standard-of-care algorithm. Throughout this review, osteosarcoma-specific evidence is distinguished from broader sarcoma or pan-solid-tumor evidence; the latter is used only as mechanistic or translational context. Proposed biomarker-treatment links are labeled according to whether support is clinical, early clinical, preclinical, or hypothesis-generating.

## 2 The osteosarcoma immune ecosystem

### 2.1 From an apparently cold tumor to a spatially compartmentalized ecosystem

Single-cell RNA sequencing has replaced the binary description of osteosarcoma as simply immune “hot” or “cold” with a more granular view. Independent studies have identified heterogeneous malignant-cell states together with variable T-cell, natural killer cell, dendritic-cell, macrophage, osteoclast-like, endothelial, and fibroblast populations (4–9). These studies consistently show that immune composition differs among patients and across disease stages. Recurrent and metastatic lesions can acquire stromal, angiogenic, and immune-remodeling programs that are not adequately represented by an archival diagnostic biopsy.

Spatially resolved analyses add a clinically important dimension. Multiplex imaging and spatial transcriptomics show that the presence of immune cells does not guarantee productive tumor contact: lymphocytes may remain peripheral, myeloid cells may occupy tumor-adjacent niches, and abnormal vascular or extracellular-matrix regions can create physical exclusion (10–12). A 2026 spatial atlas of aggressive primary and relapsed osteosarcomas identified shared immune features, systematic lymphoid exclusion, distinct myeloid patterns, and a multi-gene surface signature with potential theranostic value (11). Bulk immune scores can therefore obscure the topology that determines whether checkpoint blockade or engineered cells can access malignant compartments (Figure 1**)**.

**Figure 1:**
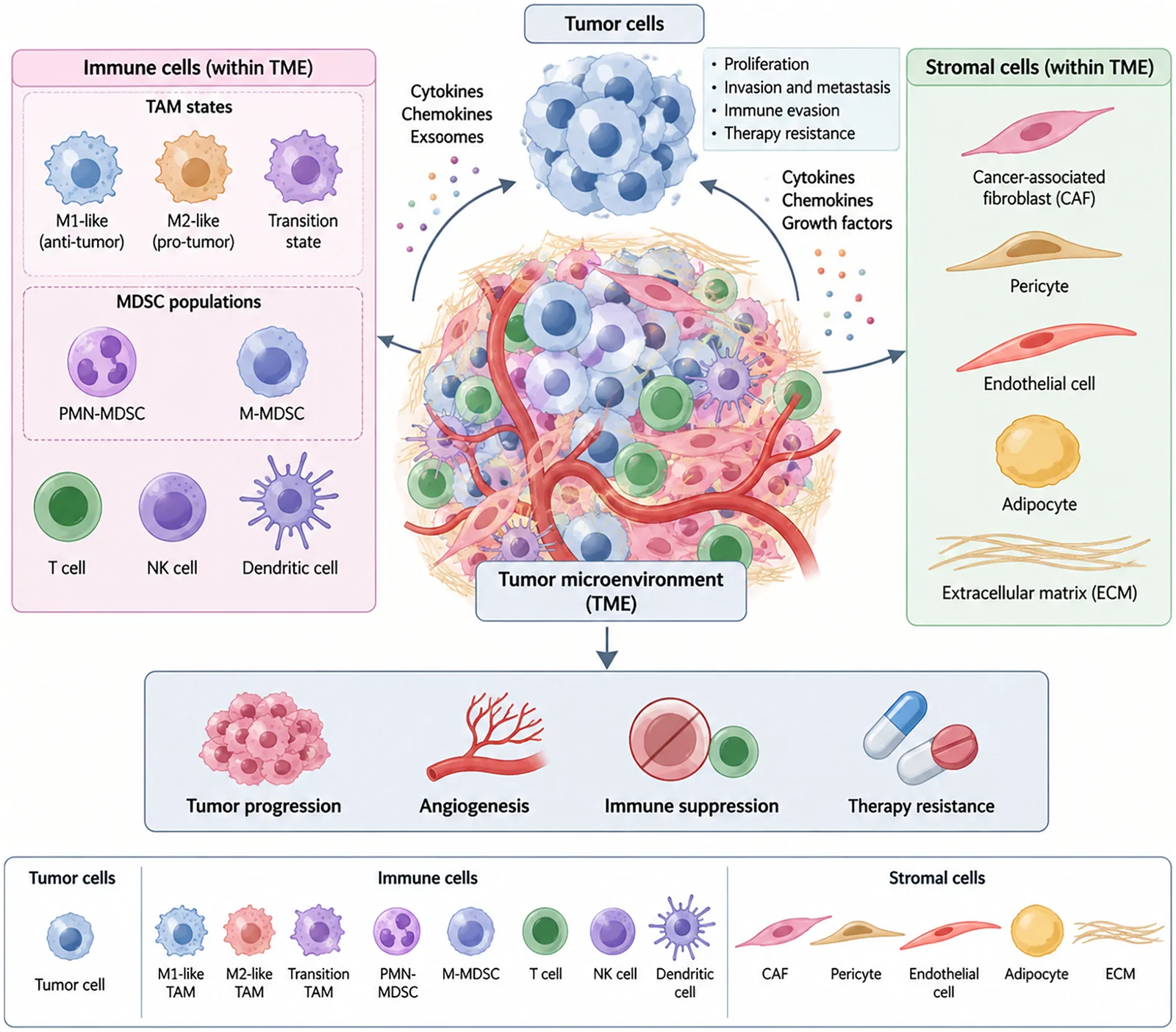
Cellular composition and functional interactions within the tumor microenvironment. The tumor microenvironment (TME) comprises tumor cells, immune cells, stromal cells, extracellular matrix, and vascular components that interact through cytokines, chemokines, exosomes, and growth factors. Within the immune compartment, tumor-associated macrophages (TAMs) are represented by M1-like, M2-like, and transitional states, while myeloid-derived suppressor cells (MDSCs) are represented by polymorphonuclear MDSCs (PMN-MDSCs) and monocytic MDSCs (M-MDSCs), together with T cells, NK cells, and dendritic cells. Stromal components include cancer-associated fibroblasts (CAFs), pericytes, endothelial cells, adipocytes, and extracellular matrix. These reciprocal cellular and molecular interactions contribute to tumor proliferation and invasion, angiogenesis, immune suppression, metastatic progression, and therapy resistance.

This spatial heterogeneity has direct sampling implications. A small core biopsy may overestimate an inflamed focus or miss an antigen-negative clone, while the primary tumor may not reproduce the immune and target profile of a lung metastasis. Biomarker programs should therefore document specimen site, treatment timing, viable-tumor content, and spatial coverage. When clinically feasible, paired primary and metastatic specimens are more informative than serial measurement of the same archival block.

### 2.2 Myeloid plasticity and the quality of lymphoid infiltration

Macrophages illustrate why cell counts alone can be misleading. In some cohorts, macrophage infiltration was associated with suppression of metastasis or favorable outcome (13), whereas other studies linked specific polarization programs and myeloid remodeling to metastatic progression (14, 15). The prognostic contribution of lymphocytes is likewise contextual: a higher intratumoral CD8/FOXP3 ratio separated survivors from non-survivors in a multicenter analysis (16), but cytotoxic cells may remain ineffective when excluded from malignant nests, deprived of costimulation, or exposed to persistent inhibitory signaling.

A useful biomarker model should distinguish inflammatory, phagocytic, osteoclast-like, SPP1-rich, ARG1-associated, and other suppressive myeloid states rather than apply a rigid M1/M2 classification. It should also record whether suppressive myeloid cells are intermingled with malignant cells, positioned around vessels, or concentrated at the invasive margin. These spatial and functional features are more actionable than total CD68-positive or CD163-positive cell counts because they can nominate myeloid reprogramming, trafficking correction, or local delivery as the relevant intervention.

Lymphoid assessment requires similar resolution. Intratumoral CD8 and natural killer cells, the CD8/regulatory T-cell balance, effector-molecule expression, and exhaustion markers should be interpreted together with tumor HLA class I and antigen-processing competence. A tumor with peripheral lymphocytes but absent HLA class I represents a different resistance state from an HLA-intact tumor with intratumoral CD8 cells restrained mainly by checkpoints. This distinction is central when deciding between HLA-dependent checkpoint strategies and HLA-independent surface-antigen targeting.

### 2.3 Checkpoint and target-antigen heterogeneity

PD-L1 expression and immune infiltration have shown prognostic associations in osteosarcoma, but results vary by assay, cutoff, sample timing, and cellular compartment (17–19). HHLA2 and other inhibitory molecules further broaden the checkpoint landscape (20). These observations argue against using PD-L1 immunohistochemistry as a stand-alone companion diagnostic. A checkpoint-rich lesion may still lack HLA class I, intratumoral CD8 cells, or adequate priming; conversely, a PD-L1-low lesion may contain surface antigens suitable for an HLA-independent cellular therapy. The marker examples summarized in Figure 1 (checkpoint molecules, antigen-presentation components, cytokines, myeloid mediators, and stromal markers) are therefore illustrative dimensions of the ecosystem rather than validated stand-alone companion diagnostics (10–20).

B7-H3 is frequently expressed in osteosarcoma and has been associated with aggressive features, making it both a candidate biomarker and a therapeutic target (21). GD2 is also widely detectable, whereas HER2 expression is often lower or more heterogeneous and depends strongly on assay methodology (22, 23). For each target, the clinically relevant question is not merely whether staining is positive, but whether membrane density, spatial coverage, metastatic-site concordance, and the differential from essential normal tissues are sufficient for the selected antibody, engager, CAR T-cell, or CAR natural killer-cell platform.

Target assessment must also anticipate evolution under selective pressure. Subclonal expression can permit immediate escape, while treatment can select antigen-low populations or alter surface availability. A single pretreatment stain is therefore least reliable when expression is heterogeneous or when the product has a steep antigen-density threshold. Multi-region profiling, quantitative flow cytometry or calibrated imaging, and post-progression reassessment should be incorporated into early trials whenever feasible.

## 3 Biomarkers for patient stratification

### 3.1 A composite tissue biomarker rather than a single stain

A practical pretreatment panel should be constructed in layers. The tumor-cell layer should quantify HLA class I and antigen-processing competence, PD-L1 and alternative checkpoints, and therapy-specific surface targets. The lymphoid layer should capture intratumoral versus peripheral CD8 cells, regulatory T cells, natural killer cells, and effector or dysfunctional phenotypes. The myeloid layer should describe macrophage and myeloid-derived suppressor cell programs. Finally, the access layer should measure fibroblast and osteoid barriers, hypoxia, vascular endothelial growth factor-related vascular states, and chemokines relevant to effector-cell trafficking.

For routine pathology, multiplex immunohistochemistry or immunofluorescence can provide an initial ecological map, while spatial transcriptomics or targeted RNA panels are most appropriate for translational trials. Decalcification method, specimen age, biopsy size, viable-tumor fraction, antibody clone, scoring compartment, and cutoff derivation must be prespecified. Whenever feasible, a contemporary metastatic or relapse specimen should supplement the primary biopsy because archival tissue may not represent the target or immune state at immunotherapy initiation.

The result should be reported as a structured phenotype rather than collapsed prematurely into one numerical score. A minimum report could state: antigen presentation intact or deficient; intratumoral lymphoid access present or absent; myeloid suppression low or high; angiogenic/extracellular-matrix exclusion low or high; and target antigen abundant/homogeneous or limited/heterogeneous. This format preserves the biological bottleneck and makes the proposed treatment action transparent. A composite score is useful only after its components, weighting, reproducibility, and treatment interaction have been prospectively validated.

### 3.2 Target-fitness biomarkers for cellular and engager therapies

Eligibility for CAR T cells, CAR natural killer cells, or T-cell-engaging antibodies requires a target-fitness assay rather than a conventional prognostic stain. At minimum, the assay should report the percentage of positive malignant cells, quantitative membrane density, intralesional heterogeneity, expression in lung metastases, and relevant normal-tissue distribution. The therapeutic threshold will differ by platform: an antibody, a high-affinity CAR, and a bispecific engager may respond differently to the same antigen density.

This distinction is illustrated by HER2. HER2 immunohistochemistry did not identify a population that benefited from adding trastuzumab to chemotherapy (23), yet HER2-specific CAR T cells demonstrated feasibility and biological activity in a sarcoma study (24). B7-H3, GD2, ALPL-1, CD166, IL-11Ralpha, and NKG2D ligands have each supported preclinical or early clinical development (25–33). Recent spatial work further suggests that multi-target surface signatures may be more robust than reliance on one antigen (11). These data support quantitative, multimodal, and preferably multi-antigen eligibility criteria, especially when clonal antigen loss is plausible.

Target fitness also includes safety and accessibility. Normal-tissue atlases, protein-level confirmation, and functional cytotoxicity across antigen-density gradients are required to define a therapeutic window. For systemic cellular therapy, eligibility should be paired with biomarkers of trafficking and expansion; for regional delivery, local target distribution and compartment-specific toxicity may be more important. Dual-target or logic-gated designs should be justified by observed coexpression patterns rather than by combining two individually attractive targets without spatial evidence.

### 3.3 Circulating and longitudinal biomarkers

Blood-based measurements can address spatial sampling error and provide early response signals. Exosomal PD-L1 and N-cadherin have been associated with pulmonary metastatic progression (34), while detectable circulating tumor DNA has correlated with inferior outcomes in osteosarcoma cohorts (35). These markers are promising for disease-burden assessment but are not yet validated predictors of benefit from a specific immunotherapy. Their value may be greatest when interpreted as serial trajectories rather than isolated baseline values.

Soluble B7-H3 highlights the interpretive complexity of liquid biomarkers. In a prospective 2026 cohort, high tissue B7-H3 was associated with unfavorable pathological response and prognosis, whereas lower baseline circulating soluble B7-H3 predicted poorer outcomes; an increase during neoadjuvant therapy was associated with progression and poor histological response (36). Tissue expression and soluble protein were not interchangeable. Biomarker development should therefore distinguish baseline prognosis, treatment-specific prediction, and on-treatment pharmacodynamic change rather than combine them under a single positive/negative label.

Longitudinal studies should prespecify collection times relative to surgery, chemotherapy, lymphodepletion, cell infusion, and radiographic assessment. Assays require analytical limits of detection, rules for low-shedding disease, and confirmation that changes precede or add information to imaging. For cellular therapies, circulating biomarker panels should be integrated with CAR expansion, persistence, cytokine profiles, toxicity, and repeat target assessment. An adaptive marker is clinically useful only when a defined result triggers a defined action. Table 1 summarizes the principal biomarker dimensions, evidence levels, and proposed therapeutic implications.

**Table 1:** Principal biomarker dimensions for osteosarcoma immunotherapy stratification.

Biomarker dimension	Biological relevance	Osteosarcoma evidence	Biomarker class	Potential implication/evidence level
Antigen presentation (HLA-I, β2M, TAP1/2)	Determines whether intracellular antigens can be presented to CD8 T cells.	HLA-I/PD-L1 patterns and immune infiltration have been described in metastatic osteosarcoma (18).	Predictive/enrichment hypothesis	Potential checkpoint/T-cell fitness marker; retrospective osteosarcoma evidence.
Lymphoid access and function (CD8, FOXP3, NK, exhaustion)	Distinguishes productive intratumoral immunity from peripheral or dysfunctional infiltration.	Spatial and immunohistochemical studies support prognostic relevance of topology and CD8/FOXP3 balance (10, 16, 17–19).	Prognostic; predictive hypothesis	Candidate enrichment dimension; not a validated companion diagnostic.
Myeloid/stromal/vascular state (TAM, MDSC, CAF/ECM, VEGF/hypoxia)	Captures immune suppression, physical exclusion, and trafficking barriers.	Osteosarcoma studies show context-dependent macrophage, stromal, and angiogenic programs (7, 10–15, 25, 31, 42).	Prognostic/pharmacodynamic hypothesis	Supports myeloid reprogramming or access-restoring combinations; mixed clinical/preclinical evidence.
Checkpoint state (PD-L1, HHLA2 and related inhibitory signals)	Reflects inhibitory signaling but not antigen presentation or immune access by itself.	Associations with prognosis are inconsistent across assays and cohorts (17–20); checkpoint trials remain weakly enriched (37–41).	Prognostic; predictive investigational	Should be combined with HLA-I and spatial immune context rather than used alone.
Surface target fitness (B7-H3, GD2, HER2, ALPL-1, CD166, IL-11R*α*, NKG2D ligands)	Defines quantitative target density, spatial coverage, and escape risk.	Target expression and therapeutic studies span osteosarcoma-specific preclinical work and limited early clinical data (21–23, 24–33).	Target-eligibility biomarker	Supports cellular/engager trials; evidence maturity differs substantially by target.
Circulating and dynamic markers (ctDNA, sB7-H3, exosomal signals)	Track disease burden and on-treatment biological change without repeated tissue sampling.	ctDNA, sB7-H3, and exosomal PD-L1/N-cadherin show prognostic or dynamic associations (34–36).	Prognostic/pharmacodynamic investigational	Useful for longitudinal monitoring; predictive utility for a specific immunotherapy is unproven.

## 4 Clinical lessons from immunotherapy studies

### 4.1 Checkpoint blockade: limited activity without validated enrichment

Checkpoint-inhibitor trials show that unselected osteosarcoma is unlikely to respond uniformly. SARC028 was a broader sarcoma trial with a small osteosarcoma cohort, in which objective activity was rare; a subsequent osteosarcoma-specific single-arm pembrolizumab study likewise showed minimal efficacy (37, 38). Pediatric and young-adult studies of nivolumab, alone or with ipilimumab, did not establish a reproducible osteosarcoma response signal (39, 40). Correlative analysis from SARC028 linked immune infiltrates with pembrolizumab response across sarcomas, but the small, heterogeneous cohorts were insufficient to define an osteosarcoma companion biomarker (41).

These negative or modest results do not prove that checkpoint pathways are irrelevant. They show that checkpoint inhibition is unlikely to overcome absent antigen presentation, lymphoid exclusion, dominant myeloid suppression, or inadequate priming by itself. A credible enrichment strategy should therefore require a composite inflamed phenotype and should test whether the biomarker modifies treatment effect, rather than merely correlates with prognosis.

Combining camrelizumab with the vascular endothelial growth factor receptor inhibitor apatinib produced clinical activity in advanced osteosarcoma, supporting a potential interaction between angiogenesis and immune exclusion (42). However, the nonrandomized design and absence of a validated biomarker prevent attribution of benefit to a specific component or subgroup. Future antiangiogenic-immunotherapy trials should prospectively assess vascular normalization, hypoxia, spatial CD8 access, and dynamic circulating markers, ideally with an appropriate comparator arm.

### 4.2 Immune-active and antigen-directed approaches

Mifamurtide remains the most clinically established immune-active agent in osteosarcoma. A Children's Oncology Group analysis reported an overall-survival benefit when muramyl tripeptide phosphatidylethanolamine was added to chemotherapy (43). More recently, immune-microenvironment gene signatures from the ISG/OS-2 trial were associated with prognosis and mifamurtide response, providing a template for treatment-specific biomarker development that now requires external and prospective validation (44). The key methodological strength is the attempt to connect a defined immune state with a defined treatment, rather than interpret the signature as a general risk score.

Antigen-directed therapy remains heterogeneous in evidence maturity. HER2-specific CAR T cells have early clinical feasibility data in HER2-positive sarcoma (24). B7-H3 has strong osteosarcoma-specific preclinical CAR T-cell evidence (25, 26) and early pan-solid-tumor clinical evidence from STRIvE-02 (32), but osteosarcoma-specific clinical efficacy has not yet been established. ALPL-1, GD2/HER2 bispecific engagement, IL-11Ralpha, NKG2D-ligand, and CD166 strategies remain predominantly preclinical in osteosarcoma (27–30, 33). Thus, antigen expression should be interpreted as target-eligibility evidence rather than proof of therapeutic benefit.

Early-phase feasibility should not be interpreted as proof of target validity in every patient. Cell dose, lymphodepletion, product phenotype, trafficking, expansion, persistence, tumor burden, and antigen density can each determine outcome. Trials should therefore couple target eligibility with serial product kinetics, tissue or imaging evidence of tumor entry, and post-treatment evaluation of antigen retention. This design can distinguish failure of the target from failure of delivery or effector fitness.

## 5 Biomarker-matched combination strategies

The purpose of biomarker stratification is to select a mechanism-matched intervention rather than to generate a prognostic score. The five ecological phenotypes proposed below are conceptual trial-enrichment states and are not clinically validated classes. They map the dominant bottleneck to a candidate strategy: inflamed/HLA class I-intact tumors to checkpoint-based approaches; myeloid-dominant tumors to myeloid modulation; angiogenic-hypoxic/lymphoid-excluded tumors to access-restoring strategies; antigen-high homogeneous tumors to target-directed cellular or engager therapy; and antigen-low/heterogeneous tumors to multi-target or logic-gated approaches.

First, an inflamed, HLA class I-intact, checkpoint-rich phenotype is the most plausible setting for checkpoint-based combinations. Entry criteria should require intratumoral rather than peripheral CD8 cells, preserved antigen presentation, and evidence that inhibitory signaling is a dominant bottleneck. PD-L1 can contribute to this profile but should not define it alone. Local radiotherapy, oncolytic or innate immune priming, and checkpoint blockade are potential combinations, but each should include a pharmacodynamic readout of improved T-cell activation.

Second, a myeloid-dominant phenotype may require macrophage or myeloid-derived suppressor cell modulation before or during checkpoint or cellular therapy. Mifamurtide-associated signatures provide a clinically anchored hypothesis (44), while all-trans retinoic acid improved CAR efficacy by reducing suppressive myeloid cells in sarcoma models (31). Biomarkers should document a biological shift in the myeloid compartment rather than assume that drug exposure equals reprogramming.

Third, an angiogenic-hypoxic and lymphoid-excluded phenotype supports combinations designed to restore access. The apatinib-camrelizumab experience provides clinical rationale (42), and engineering B7-H3 CAR T cells with chemokine receptors improved homing and antitumor activity in osteosarcoma models (25). Candidate selection should incorporate vascular endothelial growth factor-related signaling, vessel architecture, chemokine-ligand compatibility, and spatial distance between effectors and malignant cells. A trafficking endpoint is essential because radiographic nonresponse alone cannot identify whether the access barrier was corrected.

Fourth, an antigen-high and spatially homogeneous phenotype is suitable for target-directed cellular therapy or T-cell engagers. B7-H3, GD2, HER2, ALPL-1, CD166, IL-11Ralpha, and NKG2D-ligand strategies illustrate the expanding options (24–33). Platform selection should account for antigen density, normal-tissue expression, expected trafficking, and the availability of a clinically meaningful pharmacodynamic assay.

Antigen-low or heterogeneous tumors constitute a fifth phenotype in which dual- or multispecific recognition, logic-gated designs, local delivery, or sequential targeting may be needed. A multi-target strategy is especially rational when separate regions or metastases express nonoverlapping antigens (11). Conversely, adding targets without confirming coexpression may increase manufacturing and toxicity complexity without reducing escape. Serial biopsy or validated molecular imaging could determine whether target switching is required.

## 6 Discussion and future directions

The central challenge for osteosarcoma immunotherapy is not the absence of potentially actionable immune biology, but the marked heterogeneity of the tumor–immune ecosystem and the lack of validated biomarkers that can convert this heterogeneity into treatment selection. Single-cell and spatial studies consistently demonstrate substantial variation in malignant-cell states, lymphoid infiltration, myeloid composition, stromal organization, and antigen expression across patients and anatomical sites (4–12). More recent spatial profiling further indicates that lymphoid exclusion and heterogeneous surface-antigen distribution can coexist within individual tumors, providing a biological explanation for why a single biopsy or a single immune marker may inadequately represent therapeutic susceptibility (11). These findings favor a multidimensional assessment of immune access, antigen presentation, suppressive myeloid programs, and target expression rather than classification of osteosarcoma as simply immunologically “hot” or “cold.”

Clinical experience with immune checkpoint blockade illustrates this problem. Pembrolizumab and nivolumab-based studies have shown limited activity in unselected osteosarcoma populations, and available correlative analyses have not established PD-L1 or immune-cell abundance as validated predictive biomarkers (37–41). PD-L1, HHLA2, macrophage infiltration, and CD8/FOXP3-related measures may provide prognostic or biological information (13–20), but association with outcome should not be interpreted as evidence of treatment-specific benefit. The recently reported UBE2S study similarly identifies a prognostic marker associated with tumor biology and immune-related features, but it does not establish UBE2S as an immunotherapy-selection biomarker (45, 46). Likewise, Mendelian-randomization evidence linking genetically predicted circulating immune-cell traits with osteosarcoma susceptibility informs disease biology rather than therapeutic response (47). These distinctions are essential if biomarker studies are to progress from descriptive association toward clinically meaningful patient stratification

Antigen-directed cellular therapy represents a different biomarker problem. Here, the critical question is not whether a marker is prognostic, but whether the therapeutic target is expressed at sufficient membrane density, homogeneity, and anatomical accessibility to support effective recognition. HER2-specific CAR T cells have demonstrated clinical feasibility in sarcoma, whereas B7-H3, GD2, ALPL-1, CD166, IL-11R*α*, and NKG2D-ligand approaches remain distributed across preclinical and early clinical stages (24–33). The 2026 spatial osteosarcoma atlas is particularly relevant because it identified a multi-gene surface signature distributed across distinct tumor compartments, supporting the rationale for multi-target rather than single-antigen strategies in highly heterogeneous disease (11). Future cellular-therapy trials should therefore incorporate quantitative antigen-density measurements, spatial coverage, metastatic-site concordance, and post-treatment assessment of antigen loss. Failure of cellular therapy should also be interpreted in relation to trafficking, expansion, persistence, and local immune suppression rather than attributed automatically to target inadequacy

Longitudinal biomarkers may partly overcome the limitations of static tissue sampling. Circulating tumor DNA, exosomal PD-L1/N-cadherin, and soluble B7-H3 have shown associations with disease burden, metastatic progression, pathological response, or prognosis (34–36). Their current evidence, however, remains predominantly prognostic or disease-monitoring rather than predictive of benefit from a specific immunotherapy. Serial measurements are likely to be more informative than isolated baseline values because immune pressure, antigen expression, and tumor burden can change during treatment

The proposed ecological phenotypes in this review should therefore be regarded as investigational trial-enrichment categories rather than clinically validated subtypes. Their value lies in identifying the dominant therapeutic bottleneck: deficient antigen presentation, insufficient lymphoid access, suppressive myeloid states, angiogenic or stromal exclusion, or inadequate target-antigen expression. A biomarker framework becomes clinically meaningful only when the identified bottleneck is linked to a treatment capable of modifying it and when a pharmacodynamic assay confirms that the intended biological change has occurred
